# 

**Published:** 1855-01

**Authors:** 


To our Exchanges.—We have not received the "Ohio Medical & Surgical Journal," "The Journal of Pharmacy," and several others. For the benefit of publishers, we would say to them that a journal published in this city, entitled the "Medico-Chirurgical Journal," ceased to exist not quite a year since; but yet, as we learn, the former editor continues to receive exchanges. This has produced some confusion, and has been, and we fear still is the cause of mistakes, in which our exchanges are likely to take a wrong direction. We have a number of times received the exchanges due to the other periodical, but as often returned them. We repeat again, what we have several times remarked, that there is no other medical journal published in the State, except the "*Iowa Medical Journal.*"

